# Quantum machine learning with differential privacy

**DOI:** 10.1038/s41598-022-24082-z

**Published:** 2023-02-11

**Authors:** William M. Watkins, Samuel Yen-Chi Chen, Shinjae Yoo

**Affiliations:** 1 Department of Physics and Astronomy, Johns Hopkins University, Baltimore, MD 21218 USA; 2 Computational Science Initiative, Brookhaven National Laboratory, Upton, NY 11973 USA

**Keywords:** Mathematics and computing, Physics

## Abstract

Quantum machine learning (QML) can complement the growing trend of using learned models for a myriad of classification tasks, from image recognition to natural speech processing. There exists the potential for a quantum advantage due to the intractability of quantum operations on a classical computer. Many datasets used in machine learning are crowd sourced or contain some private information, but to the best of our knowledge, no current QML models are equipped with privacy-preserving features. This raises concerns as it is paramount that models do not expose sensitive information. Thus, privacy-preserving algorithms need to be implemented with QML. One solution is to make the machine learning algorithm differentially private, meaning the effect of a single data point on the training dataset is minimized. Differentially private machine learning models have been investigated, but differential privacy has not been thoroughly studied in the context of QML. In this study, we develop a hybrid quantum-classical model that is trained to preserve privacy using differentially private optimization algorithm. This marks the first proof-of-principle demonstration of privacy-preserving QML. The experiments demonstrate that differentially private QML can protect user-sensitive information without signficiantly diminishing model accuracy. Although the quantum model is simulated and tested on a classical computer, it demonstrates potential to be efficiently implemented on near-term quantum devices [noisy intermediate-scale quantum (NISQ)]. The approach’s success is illustrated via the classification of spatially classed two-dimensional datasets and a binary MNIST classification. This implementation of privacy-preserving QML will ensure confidentiality and accurate learning on NISQ technology.

## Introduction

Recent advances in machine learning (ML), particularly deep learning (DL), have been successfully applied to computer vision^1–3^, natural language processing^4^, and even toward playing the game of *Go*^5^. Notably, DL has been able to perform certain tasks with superhuman performance.

Concurrently, quantum computing machines have been introduced to the market by several tech companies. These machines are noisy and do not run in a fault-tolerant manner. Hence, they are referred to as *noisy intermediate-scale quantum* (NISQ) devices^6^. However, it has been shown that even these near-term machines can perform several calculations better than their classical counterparts^7^. Various quantum algorithms have been developed to harness the power of these near-term quantum devices, including the *variational algorithm*, which has been successful in calculating chemical ground states^8^ and optimization problems^9,10^. Other ML tasks have been shown to successfully transfer into a QML paradigm as well^11–15^.

With advances in quantum computing capabilities, a growing number of ML tasks are expected to be implemented on quantum computers^16^. Current successful ML models rely on massive datasets, and quantum machine learning (QML) is no exception. A recent study has provided a demonstration of a distributed quantum classifier, which allows one to secure the privacy of sensitive data. This is accomplished simply by allowing parties to measure or perform operations on only certain subsets of the data^17^. Most data used for building state-of-the-art ML models are collected from users. However, sensitive data, for example, personal video and voice recordings, medical records, and financial data, should never be accessible by unauthorized third-party users. Even if malicious adversaries cannot directly access the training data, they still may deduce a given data entry by attacking the trained model. One of the simplest privacy attacks is *membership inference*, in which the adversary attempts to predict if a given example was in the training set. Models will be abnormally confident on the classification of a data point in their training set, as such, the output vector from a training point will have probabilities near zero for all classes except one^18^. Even if the raw output, i.e. the confidence vector, of a model is inaccessible, *label-only membership inferences* can still break into the model by using adversarial examples. This is accomplished by determining the distance from the given point, *x*, to the decision boundary. This captures the same information as the confidence score, in that the larger the distance/the higher the confidence score, the more likely that *x* is part of the training set, so that one would say that *x* is a member of the training set, if $dist_h(x) > \tau$, where $dist_h(x)$ calculates the distance from the point to the decision boundary. The distance threshold $\tau$ for determining membership is calculated by maximizing the power of the inference on a local model’s decision boundary. In^19^, the authors demonstrate that membership inference attacks are robust against defense measures, such as confidence masking. Only differentially private training and high-level $\ell _{2}$ regularization can properly screen for (or safeguard against) such attacks.

Revealing private information is a significant problem for language models, such as GPT-2, as many are trained with either private text or sensitive public text^20^. In^18^, it showed that training data can be extracted by carefully analyzing and sampling outputs, even for models exponentially smaller than the training set. This kind of *data extraction attack* is not the only type that can result from “black box” access. *A model-inversion attack* successfully recovered images from a facial recognition algorithm in^21^ with only access to a person’s name and the confidence levels outputted from the “black box” model. Furthermore, in many applications, a hostile adversary also may have access to the model parameters. In mobile applications, the model usually is stored on the device to reduce communication with a central server^22^. *Differential privacy* (DP) is an optimization framework to address these issues.

Differential privacy involves a trade-off of accuracy and power to protect the identity of data^23^. This stems from the fact that the identity of data is masked by adding noise to the output of the model. The more noise added the stronger the privacy guarantee is, but the noise also deteriorates the machine’s ability to accurately model the label distribution. Privacy-preserving machine learning is produced by clipping the length of the loss gradients, as well as adding noise to these gradients, to reduce the effect of any one training set on the model’s parameters. This technique is applied to the training of quantum circuits in this study. Differentially private QML will allow private and efficient processing of big data. In this study, we demonstrate that the benefits of QML reduce the decrease in accuracy usually observed in privacy-preserving machine learning algorithms, i.e. differential privacy^19,24,25^. This research aims to create a hybrid quantum-classical model based on a variational quantum circuit (VQC) and train it using a differentially private classical optimizer. The classification of two-dimensional (2D) data to two classes is used to test the efficiency of the DP-VQC. As controls in the experiment, we will compare its accuracy to classical neural networks (with and without DP) and a non-private quantum circuit. Two classification tasks are used as benchmarks to compare the efficiencies of private and non-private VQCs to their classical analogs.

The novel work detailed in section “Differential privacy in quantum classification” represents the main contribution of this research, exploring how we develop a novel framework that ensures privacy-preserving QML and employ it in two benchmark examples (as follows):

- Demonstrate differentially private training on VQC-based ML models through clipping and adding noise to the gradients of the quantum circuit.
- Demonstrate that a ($\varepsilon$,$10^{-5}$)-DP VQC trains to accuracies exceeding 90% for an MNIST task with $\varepsilon$ between 0.5 and 1.0.

Section “Background” introduces the concept of differentially private ML and the required QML background. Section “Differential privacy in quantum classification” illustrates the proposed differentially private QML. Section “Experiments and results” describes the experimental settings and performance of the proposed differentially private quantum learning and is followed by additional discussions in section “Discussion”. Section “Conclusion” is the conclusion.

## Background

### Supervised learning

*Supervised learning* is an ML paradigm that learns or trains a function that maps the input to output given the input-output pairs^26^. That is, given the training dataset $\{({\varvec{x_i}},{\varvec{y_i}})\}$, it is expected that after successful training, the learned function $f_{\theta }$ is able to output the correct or approximate value ${\varvec{y_j}}$ provided the testing case ${\varvec{x_j}}$. To make the training possible, we must specify the *loss function* or *cost function* $L(\hat{{\varvec{y}}}, {\varvec{y}})$, which defines how close the output of the ML model $\hat{{\varvec{y}}} = f_{\theta }({\varvec{x}})$ is to the ground truth ${\varvec{y}}$. The *learning* or *training* of an ML model generally aims to minimize the loss function.

In classification tasks, the model is trained to output the probability of the discrete labels, i.e. targets $y_i$, given the input data ${\varvec{x}}$. For example, in computer vision applications, it is common to train ML models to classify images. The most famous example is the MNIST dataset^27^. In MNIST, there are 60, 000 images of handwritten digits of the numbers 0-9, that is, 6,000 instances of each digit. In general, a ML model is trained to approximate the probability distribution of the training data, $P(y_i \vert {\varvec{x}})$. Here, $P(y_i \vert {\varvec{x}})$ represents the conditional probability of measuring label $y_i$ given the input ${\varvec{x}}$ $\forall$ $i \in \{0,...,n_{classes}\}$. Our study is restricted to a binary classification of ’0’ and ’1’ for ease of simulating the quantum circuit.

In supervised learning, the *cross-entropy loss* is the common choice for the loss function. It can be written in the following formulation:

1 $$\begin{aligned} L(\hat{{\varvec{y}}}, {\varvec{y}}) = -\sum _{c=1}^{M} y_{o, c} \ln \left( {\hat{y}}_{o, c}\right) , \end{aligned}$$

where

- *M* = the number of classes.
- $y_{o, c}$ = the binary indicator (0 or 1) if class label *c* is the correct classification for observation *o*.
- ${\hat{y}}_{o, c}$ = the predicted probability observation *o* is of class *c*.

The loss function then is used to optimize the model parameters $\theta$. We define the hyperparameters to be the constants of an experiment that determine how the training of a model proceeds. In the current DL practice, the model parameters are updated via various gradient descent methods^28^. The “vanilla” form of gradient descent is:

2 $$\begin{aligned} \theta \leftarrow \theta - \eta \nabla _{\theta } L(f_{\theta }({\varvec{x}}),{\varvec{y}}), \end{aligned}$$

where $\theta$ is the model parameter, *L* is the loss function, and $\eta$ is the learning rate or the step-size of each updating step. Mini-batch stochastic gradient descent (SGD) simplifies ML by approximating the loss gradient when the dataset is large or when it is impractical to calculate the loss for the whole dataset at once. Suppose the training data include *N* points, then define a randomly sampled subset of points *B*. This is the mini-batch. Equation (3) approximates the gradient from the whole training set $\frac{1}{N} \sum _i \nabla _{\theta } L(f_{\theta }({\varvec{x}}_i),{\varvec{y}}_i)$ with a loss gradient calculated for a subset of the training set, the mini-batch.

3 $$\begin{aligned} {\mathbf {g}}_B = \sum _{i \in B} \frac{1}{|B|} \nabla _{\theta } L(f_{\theta }({\varvec{x}}_i),{\varvec{y}}_i), \end{aligned}$$

where *B* is the mini-batch set randomly sampled from the complete set of inputs and associated ground truth labels. This batch gradient is used in the step update rule instead of the total loss gradient $\theta \leftarrow \theta - \eta {\mathbf {g}}_B$. The batch gradient is recalculated *N*/|*B*| times per epoch, and the model parameters are updated for each gradient batch.

However, this vanilla form does not always work. For example, it may be easily stuck in local optima^28^, or it can make the model difficult to train or converge. There are several gradient-descent variants that are successfully applied in DL^28–30^. Based on previous works^31,32^, we use the RMSProp (root mean square propagation) optimizer^29^ to optimize our hybrid quantum-classical model. RMSProp is a variation of the gradient-descent method with an adaptive learning rate that updates the parameters $\theta$ as:

4a $$\begin{aligned} E\left[ g^{2}\right] _{t}&= \alpha E\left[ g^{2}\right] _{t-1}+ (1 - \alpha ) g_{t}^{2}, \end{aligned}$$

4b $$\begin{aligned} \theta _{t+1}&= \theta _{t}-\frac{\eta }{\sqrt{E\left[ g^{2}\right] _{t}}+\epsilon } g_{t}, \end{aligned}$$

where $g_t$ is the gradient at step *t* and $E\left[ g^{2}\right] _{t}$ is the weighted moving average of the squared gradient with $E[g^2]_{t=0} = g_0^2$. In this paper, the hyperparameters of the optimization technique are set for all experiments as follows: learning rate $\eta =0.05$, smoothing constant $\alpha = 0.9$, and $\epsilon = 10^{-8}$. These values were tuned using preliminary experiments.

### Quantum computing basics

Because of the power of superposition and entanglement generated by quantum gates, quantum computing can create a huge speedup in certain difficult computational tasks and afford quantum advantages to ML^33,34^. A *qubit* is the basic unit of quantum information processing that can consist of any two state system, i.e., the spin of an electron or polarization of a photon. Such a state will be written as ${|{\psi }\rangle }=\alpha {|{1}\rangle }+\beta {|{0}\rangle }$, where the probability of measuring ${|{1}\rangle }$ and ${|{0}\rangle }$ is $|\alpha |^2$ and $|\beta |^2$, respectively.

Just as all classical operations can be constructed from the set of reversible logical operations, analogous quantum operations can be formalized^33^. These operators are unitary and can be thought of as successive rotations, such that the logic operators are equivalent to quantum rotations. The basic components of quantum rotations are the Pauli matrix,

5 $$\begin{aligned} {\mathbb {I}} = \begin{bmatrix} 1 &{} 0 \\ 0 &{} 1 \end{bmatrix}, \sigma _x = \begin{bmatrix} 0 &{} 1 \\ 1 &{} 0 \end{bmatrix}, \sigma _y = \begin{bmatrix} 0 &{} -i \\ i &{} 0 \end{bmatrix}, \sigma _z = \begin{bmatrix} 1 &{} 0 \\ 0 &{} -1 \end{bmatrix}. \end{aligned}$$

With the Pauli matrix, we can define the single-qubit rotation along each of the *X*, *Y*, and *Y*-axis as follows:

6 $$\begin{aligned} \begin{aligned} R_x(\phi )&= e^{-i\phi \sigma _x/2} = \begin{bmatrix} \cos (\phi /2) &{} -i\sin (\phi /2) \\ -i\sin (\phi /2) &{} \cos (\phi /2) \end{bmatrix} \\ R_y(\phi )&= e^{-i\phi \sigma _y/2} = \begin{bmatrix} \cos (\phi /2) &{} -\sin (\phi /2) \\ \sin (\phi /2) &{} \cos (\phi /2) \end{bmatrix} \\ R_z(\phi )&= e^{-i\phi \sigma _z/2} = \begin{bmatrix} e^{-i\phi /2} &{} 0 \\ 0 &{} e^{i\phi /2} \end{bmatrix}. \end{aligned} \end{aligned}$$

The general single-qubit rotation can be constructed with two of the single-qubit rotations $R_{x}$, $R_{y}$, and $R_{z}$.

7 $$\begin{aligned} R(\phi ,\theta ,\omega ) = R_{z}(\omega )R_{y}(\theta )R_{z}(\phi )= \begin{bmatrix} e^{-i(\phi +\omega )/2}\cos (\theta /2) &{} -e^{i(\phi -\omega )/2}\sin (\theta /2) \\ e^{-i(\phi -\omega )/2}\sin (\theta /2) &{} e^{i(\phi +\omega )/2}\cos (\theta /2) \end{bmatrix}. \end{aligned}$$

For example, the quantum NOT gate also is known as the “Pauli-*X* gate,” which corresponds to a $\pi$ rotation about the *X*-axis^35^.

8a $$\begin{aligned} U_{NOT}{|{1}\rangle }= & {} {|{0}\rangle }; U_{NOT}{|{0}\rangle } = {|{1}\rangle } \end{aligned}$$

8b $$\begin{aligned} U_{NOT}= & {} e^{-i\pi \sigma _x} = \begin{pmatrix} 0 &{} 1 \\ 1 &{} 0 \end{pmatrix}. \end{aligned}$$

The true power of quantum computing stems from quantum entanglement, which can be achieved by using two-qubit quantum gates. The controlled-NOT (CNOT) gate, shown in Eq. 9, is a gate commonly used to entangle qubits. It reverses the state of second qubit if the first qubit (*control qubit*) is in the ${|{1}\rangle }$ state.

9 $$\begin{aligned} U_{CNOT} = \begin{bmatrix} 1 &{} 0 &{} 0 &{} 0 \\ 0 &{} 1 &{} 0 &{} 0 \\ 0 &{} 0 &{} 0 &{} 1 \\ 0 &{} 0 &{} 1 &{} 0 \end{bmatrix}. \end{aligned}$$

Its operation on the quantum state can be described in the following circuit diagram:
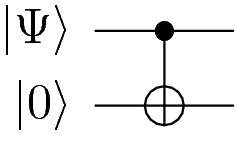


where ${|{\Psi }\rangle }$ is a single-qubit state. Concretely, if the ${|{\Psi }\rangle }$ is in the state $\alpha {|{0}\rangle }+\beta {|{1}\rangle }$, which means the system is in ${|{\Psi }\rangle } \otimes {|{0}\rangle }$, then under the CNOT operation, the state will

10 $$\begin{aligned} \begin{aligned} U_{CNOT} {|{\Psi }\rangle }\otimes {|{0}\rangle }&= U_{CNOT}\left[ \left( \alpha {|{0}\rangle } + \beta {|{1}\rangle }\right) \otimes {|{0}\rangle }\right] \\&= U_{CNOT} \left[ \alpha {|{0}\rangle } \otimes {|{0}\rangle } + \beta {|{1}\rangle } \otimes {|{0}\rangle }\right] \\&= \alpha U_{CNOT} {|{0}\rangle } \otimes {|{0}\rangle } + \beta U_{CNOT} {|{1}\rangle } \otimes {|{0}\rangle } \\&= \alpha {|{0}\rangle } \otimes {|{0}\rangle } + \beta {|{1}\rangle } \otimes {|{1}\rangle } \end{aligned}. \end{aligned}$$

The set of CNOT and single-qubit rotation operators allows for a rich group of quantum algorithms that already have been shown to be faster than their classical counterparts, for example, in factorization problems^36^ and database searching^37^. The quantum algorithm output is the observation of the final quantum state. On a real quantum computing device, the expectation values can be retrieved through repeated measurements (*shots*). In simulation, the expectation values ${\langle {0}|} U_0^{\dagger }U_1^{\dagger } \cdots U_n^{\dagger }U_n \cdots U_0U_1 {|{0}\rangle }$ can be calculated analytically. For a more detailed review of quantum computing, measurements, and algorithms, refer to^35,38,39^.

### Variational quantum circuits

In recent years, quantum computing has become feasible due to many breakthroughs in condensed matter physics and engineering. Companies, such as IBM^40^, Google^7^, and D-wave^41^, are creating NISQ devices^6^. However, noise limits the reliability and scalability in which quantum circuits can be used. For example, quantum algorithms requiring large numbers of qubits or circuit depth cannot be faithfully implemented on these NISQ devices. Because current cloud-based quantum devices are not suitable for the training described in this research, quantum circuit simulators are used^42^.

**Figure 1:**
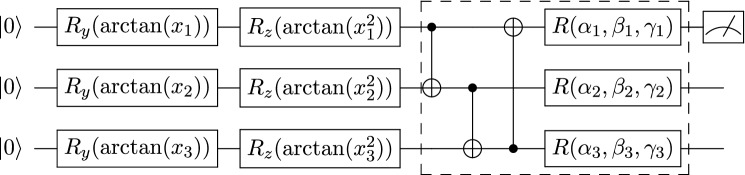
Variational quantum circuit component. The single-qubit gates $R_y(\arctan (x_i))$ and $R_z(\arctan (x_i^2))$ represent rotations along the *y*- and *z*-axis by the given angle $\arctan (x_i)$ and $\arctan (x_i^2)$, respectively. Arctan is used because the input values are not in the interval of $[-1, 1]$. The CNOT gates are used to entangle quantum states from each qubit and $R(\alpha ,\beta ,\gamma )$ represents the general single qubit unitary gate with three parameters. The parameters labeled $R_y(\arctan (x_i))$ and $R_y(\arctan (x_i^2))$ are for state preparation and are not subject to iterative optimization. Parameters labeled $\alpha _i$, $\beta _i$ and $\gamma _i$ are optimized iteratively. The dashed box denotes one layer of a quantum subcircuit. The dial to the far right represents that the circuit has one output that is the $\sigma _z$ measurement of the first qubit.

VQCs are a special kind of quantum circuit, equipped with *tunable* or *learnable* parameters that are subject to iterative optimization^11,12^. Figure 1 presents the basic components of a VQC. VQCs potentially can be robust against device noise as they can absorb the noise effects into their parameters in the optimization process^9,10^. Numerous efforts have been made to design quantum algorithms based on VQCs^9,10^, including the calculation of chemical ground states^8^ and optimization problems^43^.

Several theoretical studies have shown that VQCs are more capable than conventional deep neural networks^44–47^, in the sense that quantum models train more accurately and/or faster, when compared to classical models of comparable size. Recent results have numerically demonstrated that certain quantum architectures can perform better than their classical counterparts under specific condition. For example, quantum convolutional neural networks (QCNNs) can learn faster (with fewer training epochs) than classical CNNs and reach higher accuracies, even when the number of parameters are similar^48,49^. In^31^, a demonstration shows that a quantum long short-term memory (LSTM) can learn much faster (i.e., reach comparable accuracies with fewer training epochs) than a classical LSTM in function approximation tasks when the number of parameters are similar.

Recent studies have approached the concept of quantum supremacy with computational complexity theory^15^. Boixo et al.^50^ demonstrated that the output distributions of certain random quantum circuits can only be sampled efficiently by a direct simulation of the quantum circuit.

VQCs have been applied in several classic ML tasks, such as classification^11–14,48,49,51–57^, function approximation^11,31^, solving differential equations^58^, sequential learning^31,59,60^, and generative modeling^61–64^. Recent results have demonstrated the successful application of VQCs in the forefront of ML, for example, in metric learning^65,66^, deep reinforcement learning^32,67–69^, and speech recognition^70^.

### Differential privacy

Many technology companies collect data about the online presence of their users, and these data are shared, sometimes publicly, to use in focused marketing. This can create a breach in privacy because anonymizing data requires more than just erasing the name from each data entry^71^. Privacy also can be breached by ML models that use crowd-sourced information and data scraped from the Internet. Previous studies have shown that models memorize their training samples, and even models with millions of parameters can be attacked to output memorized data^18^.

Section “Introduction” detailed the necessity of protecting information through privacy-preserving training algorithms. In other words, anonymizing data requires more than just censoring personally identifiable information (PII) from each data entry^71^. The solution requires using DP to curtail privacy leaks.

**Figure 2:**
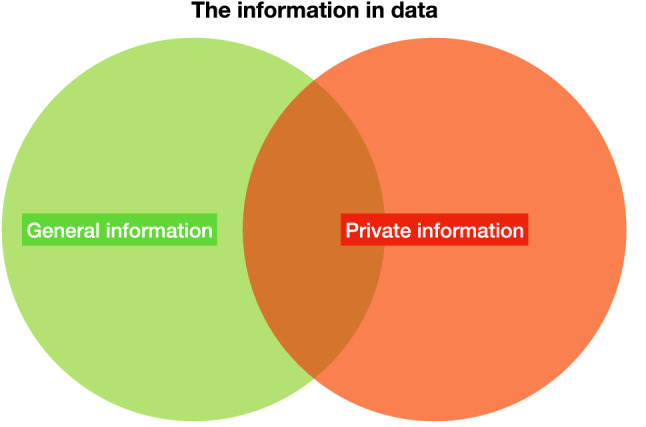
Information in data under the view of differential privacy. In a DP context, general information is that of the entire population in the data. On the other hand, private information is specific to a particular data entry.

DP is a powerful framework to restrict the information that adversaries can obtain from attacking a trained ML model, but it is not an all-powerful technique. There are two kinds of information under the perspective of DP: *general information* and *private information*. General information refers to the information that does not specify any particular data entry and can be seen as the general property of the underlying population. On the other hand, private information refers to the information that is specific to any individual data entry (Fig. 2). For a concrete example^71^, consider a study about smokers. An adversary may still learn information from the trained model, e.g., a differentially private query could show that smoking correlates to lung cancer, yet it is impossible to deduce whether or not a specific person is involved in the study. This is known as *general information*. It remains possible to deduce that an individual smoker is likely to have lung cancer, but this deduction is not due to her/his presence in the study. DP does protect an individual’s *private information*. The power of DP is that deductions about an individual cannot be influenced by the fact that the person did or did not participate in the study^71^.

We are interested in mechanisms ${\mathcal {M}}$, which are randomized algorithms. Suppose ${\mathcal {M}}$ has a domain *A* and a discrete range *B*. A randomized algorithm is defined as mapping its domain *A* to the probability space of *B*. Given an input $a \in A$, the randomized algorithm ${\mathcal {M}}$ outputs $M(a) = b$ with probability $(M(a))_b$ for each $b \in B$^71^. In DP, we seek to create a randomized algorithm, characterized by the hyperparamemers $\varepsilon$ and $\delta$, which gives roughly the same output for two similar datasets. This means an adversary cannot deduce the dataset from the output even with auxiliary information or infinite computing resources.

**Figure 3:**
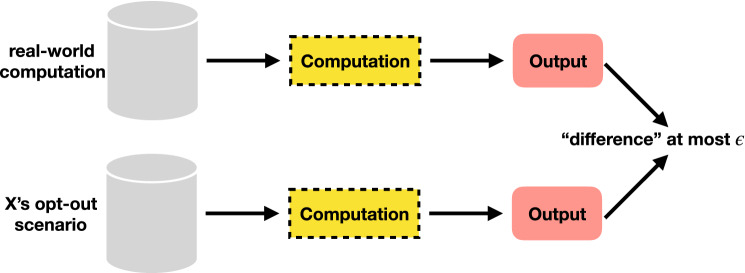
Differential Privacy.

In the context of ML, the output here is the output of a *trained model*. Figure 3 illustrates the concept of DP; the output difference between two datasets should be bounded by $\varepsilon$, for datasets differing by inclusion/exclusion of one *X*. Changing the input means the output could be very different, but DP ensures that the outputs only differ by, at most, $\varepsilon$. In other words, DP combats extraction attacks by having the output be just as likely produced from a model with or without a given training point^23^.

The domain of this randomized algorithm is the set of all possible datasets. The elements in the datasets may be discrete or continuous, as long as there is a $\ell _{1}$-norm defined, such that $\Vert x-y\Vert _1 \le 1$ implies that the *x* and *y* datasets differ by the inclusion of one element. Dwork et al. formally defines *differential privacy* in^71^ as the following: a randomized algorithm ${\mathcal {M}}$, with a domain ${\mathcal {D}}$, is $(\varepsilon , \delta )$-differentially private, if:

11 $$\begin{aligned} Pr[{\mathcal {M}}(x) \in S] \le \mathrm {exp}(\varepsilon ) Pr[{\mathcal {M}}(y) \in S] + \delta , \end{aligned}$$

for all $S \subseteq \mathrm {range}({\mathcal {M}})$ and for all $x, y \in {\mathcal {D}}$, such that $\Vert x-y\Vert _1 \le 1$, where

- ${\mathcal {M}}$ = the randomized algorithm.
- *S* = output randomized algorithm; some subset of all possible model configurations or parameters in ML context.
- *x* = a dataset used for model training.
- *y* = another dataset for model training, neighboring *x*.
- $\varepsilon$ = privacy loss for the randomized algorithm.
- $\delta$ = cutoff on DP, the percentage chance that the model does not preserve privacy.

A large $\varepsilon$ could reveal information for use in a member inference attack, because models generated from nearly identical datasets could be largely different. $(\varepsilon , \delta )$-DP is a relaxation of $\varepsilon$-DP because there is a chance $\delta$ that the privacy is broken. Furthermore, differential privacy gives the worst-case scenario privacy loss, thus a smaller $\varepsilon$ does not necessarily mean the privacy is better. This is because DP only defines an upper bound on how different the outputs can be, see Eq. (11). But there is a probability $\delta$, for which this breaks down, and then the difference of two nearly identical inputs produce significantly different outputs. Moreover, from a practical point of view, additional noise typically means that the model’s accuracy is worse. This creates a balancing act between an amount of privacy required and a level noise in the model, which does not excessively degrade the model’s accuracy.

An important characteristic in determining the effectiveness of a differentially private algorithm is the *privacy loss*. Privacy loss is defined for a given observation $\xi \in \mathrm {range}({\mathcal {M}})$, which quantifies the likeness of observing $\xi$ from ${\mathcal {M}}(x)$ versus ${\mathcal {M}}(y)$^23^.

12 $$\begin{aligned} {\mathcal {L}}^{(\xi )}_{{\mathcal {M}}(x) || {\mathcal {M}}(y)} = \ln \left( \frac{Pr[{\mathcal {M}}(x)=\xi ]}{Pr[{\mathcal {M}}(y)=\xi ]}\right) . \end{aligned}$$

A ($\varepsilon , \delta )$-differentially private algorithm is defined to have a *privacy budget* of $\varepsilon$. A practical privacy budget is heuristically defined to be under 10.0^22,72^. This creates a decent limit on the privacy leaked about a user’s data^71^.

### Differential privacy in machine learning

For ML, we can interpret the randomized algorithm ${\mathcal {M}}: A \rightarrow B$ as a training algorithm with a training set $x \in A$, which produces a model $b \in B$^22,24^. The definition of DP implies that two training sets, which only differ by the omission of a record, should be similarly likely to output a given model, i.e., the set of parameters completely describing the model.

The most basic technique to ensure DP is the *Gaussian Mechanism* as defined in^22,71,73^. Every deterministic function *f*(*d*) has a defined sensitivity $S_f = \mathrm {sup}(|f(d)-f(d^{\prime})|)$ given that $d, d^{\prime}$ are adjacent databases. Then, the Gaussian algorithm is $(\varepsilon , \delta )$-differentially private for some noise multiplier $\sigma$, such that:

13a $$\begin{aligned} {\mathcal {M}}(d)= & {} f(d) + {\mathcal {N}}(0, S_f^2 \sigma ^2 {\mathbb {I}}) \end{aligned}$$

13b $$\begin{aligned}&\delta \ge \frac{4}{5}e^{-{(\sigma \varepsilon )}^2/2.} \end{aligned}$$

There is an infinite number of pairs $(\varepsilon , \delta )$, which can be defined for a given noise multiplier $\sigma$, although usually, as in^24^, $\delta$ will be held constant in this study. Similar to the Gaussian mechanism, for a privacy-preserving machine learning algorithm, the most important technique for creating DP is to add Gaussian noise, which is then supplemented by clipping the loss gradients^22^. The gradient clip, *S*, reduces the effect any single data entry can have on the model training, making membership inference difficult. The magnitude of the gradient clip is a tunable parameter, which sets the scaling for the variance of the Gaussian noise term. In the Gaussian mechanism, the scaling of the noise term was set by the function sensitivity, $S_f$. So hyperparameters associated with these operations are the noise multiplier, $\sigma$, and a cutoff for the $\ell _{2}$ norm, *S*^22,24^. After calculating the gradients, if the batch gradient has an $\ell _{2}$ norm greater than the cutoff, it is scaled down to have a norm equal to the cutoff. After clipping the gradient, the gradient for the mini-batch has Gaussian noise added with a standard deviation equal to the $\ell _{2}$ norm cutoff, i.e., *S*, multiplied by the noise factor, $\sigma$,

14a $$\begin{aligned}&{\mathbf {g}}_B \leftarrow \left[ {\mathbf {g}}_B*\mathrm {min}\left( 1, \frac{S}{||{\mathbf {g}}_B||}\right) + {\mathcal {N}}(0,\sigma ^2 S^2 {\mathbb {I}} ) \right] \end{aligned}$$

14b $$\begin{aligned}&\sigma \ge c_2 \frac{q\sqrt{n_e/q\ln (1/\delta )}}{\varepsilon } \end{aligned}$$

14c $$\begin{aligned}&q = |B|/N \end{aligned}$$

where *q* is the ratio of the batch size, |*B*|, to the number of training samples, *N*, and $n_e$ is the number of epochs. Like the Gaussian mechanism and Eq. (13a), an implicit relationship exists, given in Eq. (14b), determining the privacy loss, $\varepsilon$, from $\delta$ and $\sigma$. This modification to the optimizer algorithm can be applied to any ML algorithm (SGD, Adam, RMSprop, etc.). The DP-SGD algorithm is based on the techniques from^22,73^. Details on the privacy loss calculation and ones specific to the software package used in this study to implement DP, PyVacy, are available in the appendix.

### Power of quantum models

**Figure 4:**
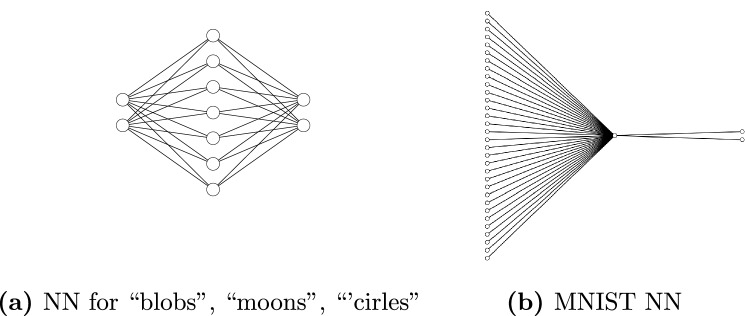
Architecture for the classical neural networks used controls. For (**a**) the left layer is the 2D input and for (**b**) the left layer is the 1024 values from the padded MNIST grayscale images. While the spatial classifier has seven in the hidden layer, the MNIST classifier has only one. The number of nodes is set such that they can be compared to the variational quantum circuits, VQCs, fairly. The right layers have two nodes each, since both tasks are binary classifications. After being normalized by the log-loss function, the output is a vector of the probability of being in each class given the input. For the sake of readability, only 32 of the 1024 input nodes are shown for the MNIST NN.

An important concept in machine learning is the power of the model, that is, the ability of the model to learn different and complex relations between the input and output datasets. Multiple studies have discussed and observed a numerical advantage in quantum neural networks over classical ones, in terms of accuracy, loss and convergence^31,48–50^. On the other hand, Wright et al. use a metric known as *memory capacity* to express the power of the model, and the authors find that, for classically parametrized QNNs, there is no advantage compared to NNs with the same number of parameters^74^. Abbas et al. analyze the power of QNNs from the perspective of information geometry. They attempt to quantify the potential advantages of QNNs by using the concepts of *effective dimension*, which is calculated from the *Fisher information* metric. The *Fisher information* metric measures the amount of information the observation of the random variables, ${\mathcal {X}}$, carries about the parameters, $\theta _i$, that characterize the distribution of ${\mathcal {X}}$. They demonstrate that the *Fisher information* spectrum is more spread out in their quantum models than in their classical model. The average *Fisher information* spectrum for the ’hard encoding’ QNNs’ has more larger eigenvalues, compared to the spectrum of classical NNs, so the training will theoretically encounter less barren plateaus and be more efficient than its classical counterpart. The study also investigates the *effective dimension* against number of data. The ’hard encoding’ QNNs has the highest *effective dimension*, which also converged to *d*, the rank of the *Fisher information* matrix the fastest. The authors further demonstrate that the *effective dimension* of the classical NNs converges slower, because the NNs have a more degenerate average *Fisher information* spectrum spectrum^47^. This motivates our study to hypothesize that quantum neural networks would also perform better in a privacy-preserving regime compared to classical networks (Fig. 4).

The fact that quantum neural networks project classical inputs into a Hilbert Space presents the possibility of a lower privacy loss in QML models. Liao et al. prove that the predictive confidence difference in any quantum protocol is upper bounded by the difference in the trace norm of the input density matrices, up to a constant^75^. This leads one to believe that the standard privacy budget calculation, as used in this study, may be a more generous bound for differentially private quantum classifiers than for differentially private classical classifiers. For the sake of comparison and simplicity, we only calculate the privacy budget as defined in^22^. We leave the development of a tighter upper bound on privacy loss in trained quantum circuits to a future study.

Another boon to improving privacy bounds on quantum protocols is the inherent presence of noise in noisy intermediate-scale quantum (NISQ) devices. For instance, Du et al. discuss how adding depolarizable noise to a quantum circuit imposes differential privacy on the model, providing robustness against adversarial examples^76^. However, the simulated quantum circuits used in the models of this study are ideal, so there is no inherent noise in the circuit. Furthermore, this study aims to provide privacy budget guarantees by adding classical noise to the training protocol instead of adding noise to the model, so we do not utilize depolarizing noise in our effort to provide differential privacy to our quantum circuits.

## Differential privacy in quantum classification

**Figure 5:**
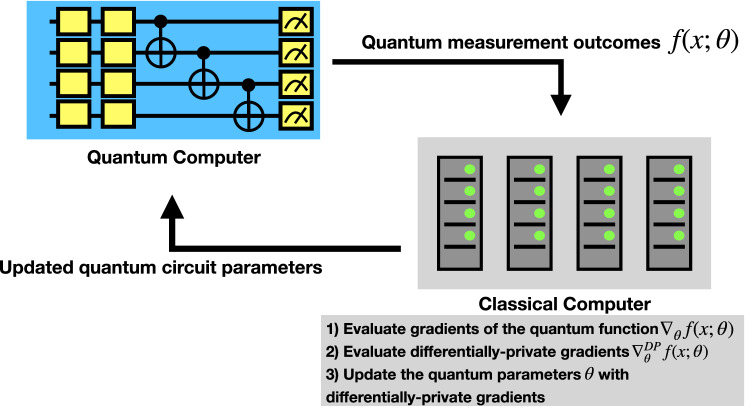
Differential Privacy in Quantum Machine Learning. In the proposed framework, the outputs from the quantum circuit are processed on a classical computer. The gradients of the quantum function $\nabla _{\theta }f(x;\theta )$ and the differentially private gradients $\nabla _{\theta }^{DP}f(x;\theta )$ are calculated. The quantum circuit parameters are updated according to the differentially private gradients and fed back to the quantum computer.

In this work, we propose a hybrid quantum-classical framework interfacing the differentially private classical optimization algorithms with VQC-based QML algorithms. In a hybrid quantum-classical model architecture, the quantum circuits are used to generate the output, mostly in the form of quantum measurement. The measured expectation values then can be used to evaluate the *loss function* on a classical computer, which then will be used to evaluate the model’s performance and adjust the circuit parameters. The updated circuit parameters are then fed back to the quantum computer. This iterative process gradually *trains* the quantum circuit to achieve the desired results. The DP training in such a hybrid quantum architecture exists in the gradient calculation process, which is on the classical computer. Figure 5 presents the proposed scheme.

### Quantum encoding

A quantum circuit operates on the quantum state. To make QML useful, the first step is to encode the classical data into a quantum state.

#### Amplitude encoding

*Amplitude encoding* is a technique to encode the classical vector $(\alpha _{0} \cdots \alpha _{2^n-1})$ into an *n*-qubit quantum state ${|{\Psi }\rangle } = \alpha _{0}{|{00\cdots 0}\rangle } + \cdots + \alpha _{2^n-1}{|{11\cdots 1}\rangle }$. The advantage of using this encoding method is that it is possible to significantly reduce the number of qubits and potentially the number of parameters of the quantum circuit. An *N*-dimensional input vector would require only $\log _{2}N$ qubits to encode. Refer to^77,78^ for details regarding this encoding procedure.

#### Variational encoding

In *variational encoding*, the input values are used as the quantum rotation angles. A single-qubit gate with rotation along the *j*-axis by angle $\alpha$ is given by:

15 $$\begin{aligned} R_j(\alpha )=e^{-i\alpha \sigma _j/2}=\cos \frac{\alpha }{2} I-i\sin \frac{\alpha }{2}\sigma _j, \end{aligned}$$

where *I* is the identity matrix and $\sigma _{j}$ is the Pauli matrix with $j = x, y, z$. In this work, given a vector input $x_N$ with *N* dimensions, we rotate each qubit by $R_i(x), i \in [0,N)$:

16 $$\begin{aligned} \begin{aligned} R_i(x)&=e^{-i\beta _i\sigma _z/2}e^{-i\alpha _i\sigma _y/2}, \\ \alpha _i&= \arctan (x_i); \beta _i = \arctan (x_i^2). \end{aligned} \end{aligned}$$

Each single-qubit state is initialized by rotations in the *y*-axis then in the *z*-axis. This allows our inputs, $x \in X$, to be encoded into a quantum state of *N* qubits. Figure 1 depicts this particular encoding scheme. For a detailed review of different quantum encoding schemes, refer to^77^.

### Quantum gradients

Modern DL practices heavily depend on gradient-based optimization methods. Classically, the gradients of DL models are calculated using *backpropagation* methods^79^. In QML, the corresponding method is the *parameter-shift rule*, which can calculate the analytical gradients of quantum models^11,42^. This is similar to the derivative-free technique developed in^80^. The Pennylane quantum software library uses the parameter shift to calculate the gradients of the quantum circuit.

For the parameter-shift rule, knowledge of certain observables are given. A VQC’s output can be modeled as a function of its parameters $f(x; \theta )$ with parameters $\theta$. Then, in most cases, the partial derivative of the VQC, $\nabla _{\theta } f(x; \theta )$, can be evaluated with the same quantum circuit only with the parameters shifted^11^. We illustrate the procedure as follows: consider a quantum circuit with a parameter $\theta$, and the output can be modeled as the expectation of some observable, e.g., *B* for some prepared state ${|{\psi }\rangle }=U(\theta )U_0(x){|{0}\rangle }$ or $f(x; \theta ) = {\langle {0}|}U^{\dagger }_0(x)U^{\dagger }(\theta ){\hat{B}}U(\theta )U_0(x){|{0}\rangle }$. This is simplified by considering the first unitary operation as preparing the state ${|{x}\rangle }$ and the other unitary operators as a linear transformation of the observable, $U^{\dagger }(\theta ){\hat{B}}U(\theta ) = {\mathcal {M}}_{\theta } ({\hat{B}})$.

17a $$\begin{aligned} f(x; \theta )= & {} {\langle {x}|}{\mathcal {M}}_{\theta } ({\hat{B}}){|{x}\rangle }, \nonumber \\ \nabla _\theta f(x; \theta )= & {} {\langle {x}|} \nabla _\theta {\mathcal {M}}_{\theta } ({\hat{B}}){|{x}\rangle }, \end{aligned}$$

17b $$\begin{aligned} \nabla _\theta {\mathcal {M}}_{\theta } ({\hat{B}})= & {} c[{\mathcal {M}}_{\theta +s} ({\hat{B}}) - {\mathcal {M}}_{\theta -s} ({\hat{B}})]. \end{aligned}$$

It can be shown that a finite parameter, *s*, exists, such that the Eq. (17b) stands^11^. This implies that the quantum circuit can be shifted to allow for a calculation of the quantum gradient with the same circuit.

Now that DP and our VQC architecture are introduced, we unveil our differentially private optimization algorithm—the first of its kind to ensure privacy-preserving QML. Our differentially private optimization framework starts by calculating the quantum gradient using the parameter shift rule. Next, we apply Gaussian noise and clipping mechanisms to this gradient, $\nabla _{\theta } f(x; \theta )$. The differentially private gradient, $\nabla _{\theta }^{DP} f(x; \theta )$, now is used in the parameter update step instead of the non-private gradient. This parameter update rule can be SGD, adaptive momentum, or RMSprop. In this study, we solely use RMSprop to update parameters.

18 $$\begin{aligned} \nabla _{\theta }^{DP} f(x; \theta ) = \left[ {\langle {x}|} \nabla _{\theta } {\mathcal {M}}_{\theta } ({\hat{B}}){|{x}\rangle }*\mathrm {min}\left( 1, \frac{S}{|{\langle {x}|} \nabla _{\theta } {\mathcal {M}}_{\theta } ({\hat{B}}){|{x}\rangle }|}\right) + {\mathcal {N}}(0,\sigma ^2 S^2 {\mathbb {I}} ) \right] , \end{aligned}$$

where ${\mathcal {M}}_{\theta } ({\hat{B}})$ is defined in Eq. (17a) and $S, \sigma$ are the hyperparameters implicitly defining the level of privacy $(\varepsilon , \delta )$. This novel framework seamlessly incorporates privacy-preserving algorithms into the training of a VQC, ensuring $(\varepsilon , \delta )$-differential privacy. In this work, we choose the standard classification task to demonstrate the proof-of-concept result. However, the proposed framework is rather generic and can be applied to any hybrid quantum-classical ML scenarios.

## Experiments and results

To demonstrate the hypothesized quantum advantage, this study compares differentially private VQCs (DP-VQCs) to non-private VQCs, as well as private and non-private neural networks. We also illustrate the efficacy of our differentially private QML framework. Two different types of classifications will be investigated as benchmarks: 1) labeling points in a 2D plane and 2) a binary classification from an MNIST dataset, differentiating between the ‘0’ and ‘1’ digits. The 2D datasets are standard benchmarks from scikit-learn^81^ that are useful in QML because the inputs are low dimensional, thus easy to simulate on classical computers^34^. Meanwhile, the MNIST dataset is used to study the performance of the proposed model with larger dimensional inputs.

We implement the model with several open-source software packages. The high-level quantum algorithms are implemented with PennyLane^82^. The quantum simulation backend is Qualacs^83^, which is a high-performance choice when the number of qubits is large. The hybrid quantum-classical model is built with the PyTorch interface^84^. For differentially private optimization, we employ the PyVacy package^85^.

The experiments are characterized by the hyperparameters of the neural network training process: the optimizer, number of epochs, number of training samples, learning rate, batch size, momentum, and weight penalty. When differentially private optimizers are used, the additional hyperparameters needed are the $\ell _{2}$ norm clip, noise multiplier, number of iterations, and $\delta$. After preliminary experiments, the RMSprop optimizer was selected for use in all of the experiments presented in this paper. Most of the model’s hyperparameters are the same for both the MNIST and scikit 2D set classification tasks. Table 1 depicts the hyperparameters used in this study; the learning rate is set to 0.05, while the proportion of training and testing is 60% and 40%, respectively. In addition, the batch size used is 32 with a momentum value of 0.5, but no weight regularization is used.

All tasks are classifications, so cross-entropy is used as the loss function for all training. It is common to choose $\delta \in O(1/n)$ for *n* samples, because a mechanism can satisfy $(0,\delta )$-differential privacy for larger $\delta$, but still breach privacy for $n\delta$ data points based on Eq. (11)^23,71^. We set $\delta$ to be $10^{-5}$ for the entire study, so $\varepsilon$ is determined implicitly by Eq. (14a) and the hyperparameters: *S*, $\sigma$, and $\delta$.

**Table 1:** Hyperparameters chosen for non-private and differentially private classifiers.

Exp	LR	Mom.	Batch	$\ell _{2}$ Clip	Noise Mult.	# of Iter.	$\delta$
Non-private	0.05	0.5	32	n/a	n/a	n/a	n/a
DP	0.05	0.5	32	1.0	varies	5	$10^{-5}$

The neural networks and VQCs use the same hyperparameters for both classification tasks. The learning rate (LR) is the same across all experiments. Different noise multipliers are used to compare differentially private networks. The “varies” noise parameter means that multiple values of noise have been used in the DP-neural network and DP-VQC experiments. $\varepsilon$ also varies among DP experiments as it directly depends on the noise multiplier. The last four hyperparameters are applicable only with differentially private optimizers.

As part of the investigation into differentially private QML, classical and quantum classifiers are compared. For both the MNIST and 2D classifiers, the quantum circuit has two modules that contain the parameters for the unitary transforms comprising the two quantum subcircuits.

### Two-dimensional mini-benchmark datasets

Three datasets of 2D classification from scikit-learn are considered. 200 points are generated for the “blobs” and “moons” dataset, and the data is divided into training and testing sets with the proportions being 60% and 40%, respectively. Different datasets are used because the decision boundary between the two classes is increasingly nonlinear and more difficult to classify. Thus, they make good benchmarks for DP training. The “circles” dataset was generated with 1000 points instead of 200 based on preliminary experimentation with privacy-preserving classification. The leftmost plots of Figure 10 display the input sets, which are named “blobs,” “moons,” and “circles” based on the shapes they form. The more transparent points are those not part of the training, but instead used for testing the model’s accuracy.

As a baseline for the study, Figure 4a illustrates the classical neural network written with two classical layers. The classical classifier uses $\tanh$ as the activation function after each layer and softmax at the end of the calculation. The neural network has Xavier weight initialization. The linear layers sizes are such that the number of total trainable parameters in the quantum classifier is 66%, while the number of trainable parameters in the classical classifier is 24 for the VQC and 36 for the neural network.

**Figure 6:**
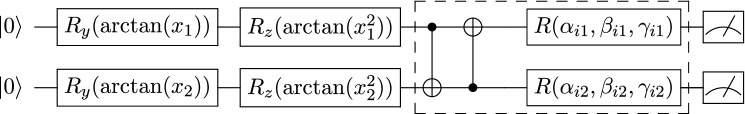
First quantum circuit block for 2D classification. The single-qubit gates $R_y(\arctan (x_i))$ and $R_z(\arctan (x_i^2))$ represent rotations along the *y*-axis and *z*-axis by the given angle $\arctan (x_i)$ and $\arctan (x_i^2)$, respectively. The state is prepared with *variational encoding*. The dashed box denotes one layer of a quantum subcircuit that is repeated twice. At the end of this circuit, two qubits are measured, and the *Z* expectation values are calculated. The output from this circuit is a 2D vector.

**Figure 7:**
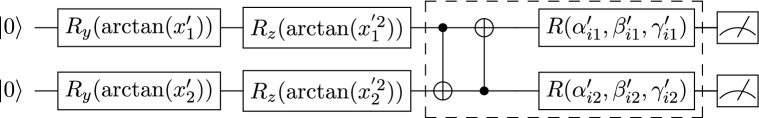
Second quantum circuit block for 2D classification. The parameters labeled $R_y(\arctan (x^{\prime}_i))$ and $R_y(\arctan (x_i^{\prime 2}))$ are for state preparation. $x^{\prime}_1$ and $x^{\prime}_2$ are the outputs of the first circuit block. The dashed box denotes one block of a quantum circuit that is repeated twice. At the end of this circuit, two qubits are measured, and the *Z* expectation values are calculated. The output from this circuit is a 2D vector. In the context of cross-entropy loss, the outputs will be interpreted as the probability that the 2D point belongs to class one or two, respectively.

The VQC to classify the 2D test set consists of two successive quantum subcircuits (Figs. 6, 7). Each quantum subcircuit has two wires, while each unitary transform can be thought of as rotations on each qubit. Thus, each subcircuit is parameterized by 12 Euler angles or parameters because there are two layers of transforms per subcircuit. The angles are initialized on a normal distribution with mean 0, standard deviation 1.0, and then scaled by 0.01.

**Table 2:** Accuracies of differentially private neural networks and variational quantum classifiers after 30 epochs for 2D input sets: “blobs,” “moons,” and “circles.”

$\varepsilon$	$\delta$	NN-blobs	VQC-blobs	NN-moons	VQC-moons	NN-circles	VQC-circles
Non-DP	n/a	1.00	0.96	0.99	0.87	1.00	0.97
1.628	$10^{-5}$	0.98	0.92	0.91	0.88	0.88	0.998
0.681	$10^{-5}$	0.98	0.92	0.87	0.85	0.86	1.00

The quantum classifier can achieve DP with more accuracy for the “circles” set. For the “blobs” and “moons,” the quantum and classical classifier has nearly the same accuracy under a given level of DP.

Table 2 summarizes the key results from the 2D classification experiments. Three different levels of privacy have been investigated non-private, $(1.628, 10^{-5})$-DP, and $(0.681, 10^{-5}))$-DP on three different input sets “blobs,” “moons,” and “circles.” For most pairs of model architecture and input set, the differentially private result has a lower accuracy than the non-private one. There are three exceptions though; the VQC “moon” classifier is more accurate with $(1.628, 10^{-5})$-DP than without, and the VQC “circle” classifier is more accurate at both levels of differential privacy than the non-private classifier.

**Figure 8:**
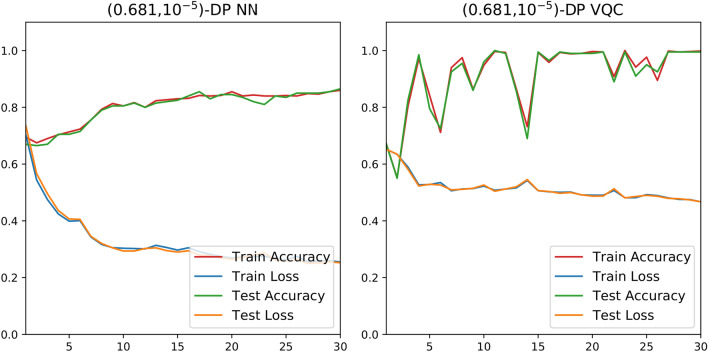
Results for differentially private NN and variational quantum classifier of “circles” with 1000 samples, a learning rate of 0.05, and RMSprop optimizer.

As detailed in Table 2, the classical and quantum classifiers are almost equally successful for the “blobs” and “moons” sets. On the other hand, Fig. 8 demonstrates that the DP-VQC affords superior performance for the “circles” set as the quantum classifier is 14% more accurate than the DP-neural network. The last two columns of Fig. 10 depict the decision boundary and accuracy of privacy-preserving $(0.681, 10^{-5})$-differentially private classical neural networks and VQCs.

**Figure 9:**
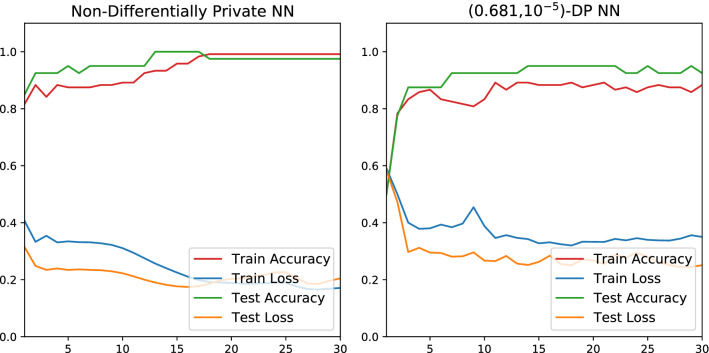
Results for “moons” classical classifier with 200 samples in total, 120 for training and 80 for testing, a learning rate of 0.05, and RMSprop optimizer with and without DP.

**Figure 10:**
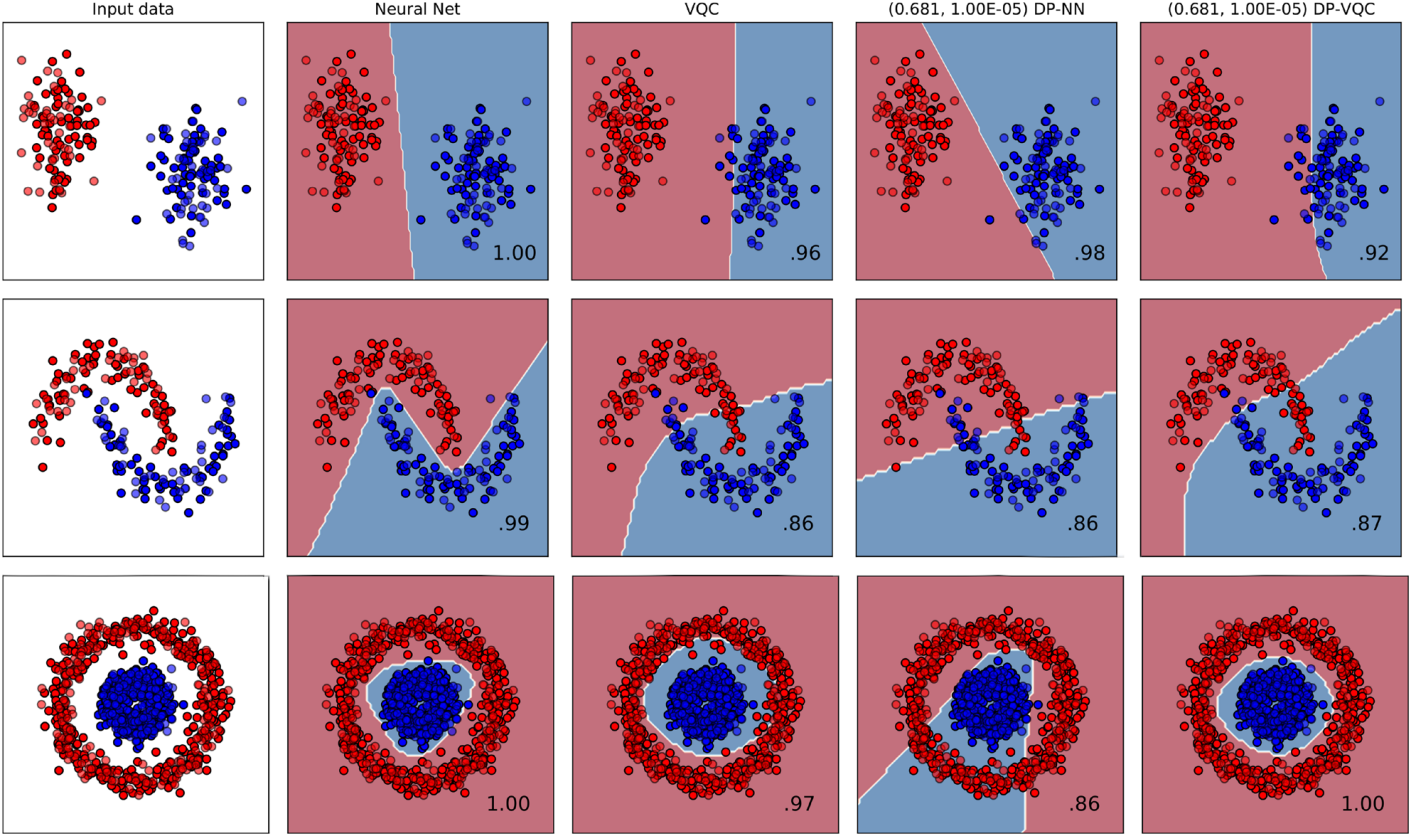
Results from the 2D ML experiments. The first column shows the three input sets, which are the “blobs”, “moons”, and “circles” datasets going down. The subsequent columns show different models tasked with classifying and learning the decision boundary. The array of plots illustrates the decision boundaries formed by the different models. The solid points are those used in training, and the transparent ones are part of the testing set. Total accuracy after 30 epochs is displayed on the lower right of each plot. The first two datasets each contained 200 samples; the last dataset, i.e. “circles”, contains 1000 samples.

The comparison of Fig. 9 to Fig. 8 demonstrates that the neural network’s efficiency under DP training differs for different datasets. For the “moons” input set, the accuracy degradation from DP is somewhat significant at 10%. Yet with the “circles” set, the accuracy actually increases 3%. Figure 8 illustrates that the private neural network trains more smoothly than the DP-VQC, but also that it reaches a final accuracy 14% lower than the DP-VQC’s final accuracy (Fig. 10).

### MNIST binary classification

The MNIST classification task is prepared similarly to the 2D classification problem. Because of the computational complexity of simulating large quantum systems, the problem is reduced to a binary classification of distinguishing the handwritten digits of ‘0’ and ‘1.’ The digits are grayscale images with a total of 784 pixels. The variational quantum classifier uses *amplitude loading* (described in section “Amplitude encoding”) to compress the number of inputs to fit within 10 qubits. Therefore, the 784 inputs are padded with additional zeros to make the inputs 1024 dimensional. Next, *amplitude loading* transforms the 1024 pixels into a 10-qubit quantum state for operating the variational quantum classifier.

The MNIST neural network uses the same padded 1024 pixels as an input. This NN has one hidden layer with one node, and an output layer with two nodes, as depicted in Fig. 4b. Hence, the classical model has 1029 parameters divided between the two weight matrices and biases. The design for this classical benchmark aims to limit the number of parameters for fair comparison to the quantum model. Even with only a single node in the hidden layer, the classical MNIST classifier will have four times the number of parameters of the quantum classifier.

**Figure 11:**
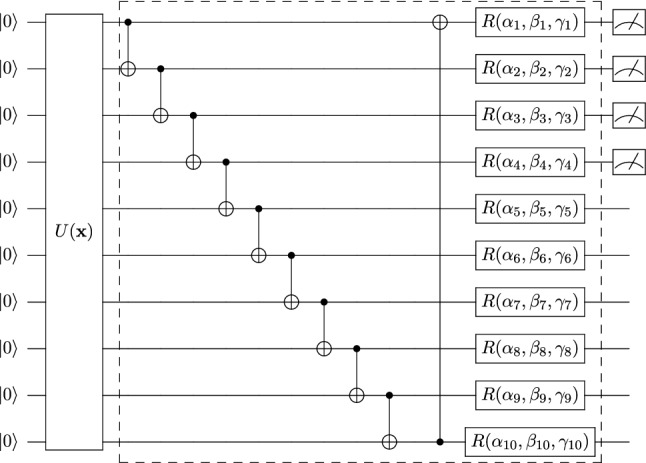
First quantum circuit block for MNIST classification. The first VQC block encodes the MNIST image. The 1024-dimensional vector is encoded via amplitude encoding into a $\log (1024)$, i.e., 10-qubit state. $U({\mathbf {x}})$ denotes the quantum algorithm for amplitude encoding as explained in^77,78^. $\alpha _{i}$, $\beta _{i}$, and $\gamma _{i}$ are the parameters to optimize. The dashed box denotes one block of a quantum circuit that is repeated eight times. Thus, there are $30 \times 8 = 240$ parameters to the circuit block. The dial to the far right represents that the circuit has four outputs. The expectation of $\sigma _z$ is measured on four qubits. The output becomes the input for the next circuit block.

**Figure 12:**
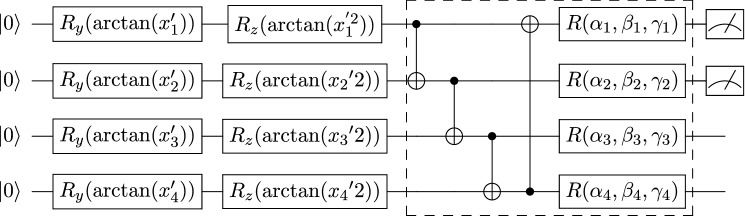
Second quantum circuit block for MNIST classification. The second subcircuit uses *variational encoding* to encode the output from the first block to be the input for this subcircuit. $\alpha ^{\prime}_{i}$, $\beta ^{\prime}_{i}$, and $\gamma ^{\prime}_{i}$ are the parameters to optimize. The dashed box denotes one block of a quantum circuit that is repeated four times. There are $12 \times 4 = 48$ parameters to the circuit block. The dial to the far right represents that the circuit has four outputs, and the expectation of $\sigma _z$ is measured on two qubits. In the context of cross-entropy, the outputs will be interpreted as the probability that the image is of a ‘0’ or a ‘1,’ respectively.

**Figure 13:**
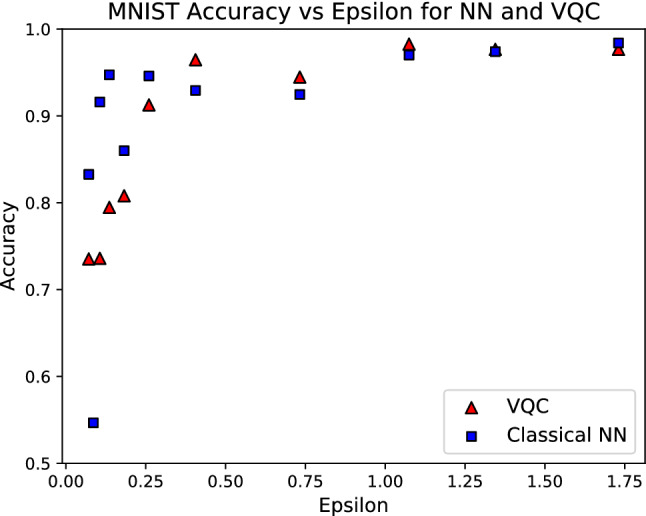
Accuracy of DP classifiers after 30 epochs plotted against their $\varepsilon$ for a MNIST binary classification of the handwritten digits ‘0’ and ‘1.’ With only 28% the number of parameters in the NN, the quantum classifier is at least as accurate as the classical classifier for all investigated DP levels.

The quantum classifier has two quantum subcircuits. The first has 10 inputs, eight layers of unitary transforms, and four outputs (Fig. 11). Each qubit has a tunable unitary transform per layer, so there are $8 \times 10 \times 3 = 240$ parameters in the first subcircuit. The second subcircuit has four inputs, two outputs, and four layers (Fig. 12), so it has $4 \times 4 \times 3 = 48$ tunable parameters associated with the rotations of quantum bits. Consequently, the VQC has 288 parameters. Importantly, this represents roughly only a quarter (27.99% exactly) of the number of parameters associated with the analogous classical neural network used for the same classification task. The MNIST results are summarized in Table 3. Multiple levels of privacy are created by iterating the noise multiplier from 1.0 to 5.0. The privacy budget for such noise is between 1.73 to 0.07, respectively. Figure 13 and Table 3 exemplify that the accuracies of both neural networks and VQCs decrease as $\varepsilon$ decreases. This emphasizes the trade-off between utility and privacy in differentially private algorithms.

**Table 3:** Results from binary MNIST classification of ’0’ and ’1’ digits.

$\epsilon$	$\delta$	Classical NN	VQC
1.73071508	$10^{-5}$	0.984	0.97666667
1.3448161	$10^{-5}$	0.974	0.97666667
1.07469683	$10^{-5}$	0.97	0.98266667
0.73250501	$10^{-5}$	0.92466667	0.94466667
0.40585425	$10^{-5}$	0.92933333	0.96466667
0.25998742	$10^{-5}$	0.946	0.91266667
0.18230998	$10^{-5}$	0.86	0.808
0.13604452	$10^{-5}$	0.94733333	0.79466667
0.10626109	$10^{-5}$	0.916	0.736
0.07149769	$10^{-5}$	0.83266667	0.73533333

Accuracies of differentially private neural networks and variational quantum classifiers after 30 epochs. The private quantum classifier is more accurate and successful for $\varepsilon$’s between 0.41 and 1.34.

## Discussion

### Potential applications of private QML

Differentially private data are becoming more critical because larger models have been shown to memorize more data, e.g., language models^18^. One of the latest state-of-the-art language models, GPT-2, has 1.5 billion parameters and was found to memorize 18 times more information when compared to a 124 million parameters language model. The aforementioned study demonstrates that training data extraction attacks are practical. This necessitates an implementation of a privacy-preserving algorithm, i.e., DP, to curtail memorization and data extraction attacks.

This study has presented the implementation and a successful proof-of-concept application of DP to QML models. The employment of differential privacy can be extended to a myriad of applications that require privacy-preserving learning and the expressibility of quantum models. This method of privacy-preserving training has an ease of deployment, because it is straightforward to add in the private mechanism, which only affects the gradients during the training process. That is, the gradients are clipped to some value, *S*, and Gaussian noise is added equally in each gradient direction, which ensures differential privacy with a privacy budget of *varepsilon*. As mentioned before, *varepsilon* is calculated from *sigma* and *delta* as illustrated in Eq. (14b). One potential application is facial recognition, since these models must train on thousands faces, whose identities are not protected from^20^. Therefore, this field would intrinsically benefit from DP, and QML could create even more accurate predictions based on our empirical results in this study and^53^. QCNN would be another logical application of a private QML algorithm as QCNNs already are being investigated with the MNIST and other benchmarks^49,55,86^. As such, it is expected that a privacy-preserving framework would benefit these application scenarios, including QCNN. Our results show that the private VQC distinguishes between the ‘0’ and ‘1’ digits with an accuracy exceeding 90% and outperforming the classical NN, for privacy budgets greater than 0.25. On the other hand, for budgets less than 0.25, the classical classifier is more accurate.

With recent QML developments impacting a spectrum of applications, such as speech recognition^70^, quantum recurrent neural network (QRNN) and quantum LSTM for sequential learning^31,59,60^, and even certain emerging applications in medical imaging^87^, we expect the framework described by this work would be of benefit to these new scenarios as well.

An important point to consider is the limitation of extending these results to real-world quantum devices. High-dimensional input, such as MNIST, takes an extremely long time to run on a cloud-based quantum computer. However, it is possible to run. One limitation of this study is that the VQCs are simulated with noise-free quantum computers. A future study could investigate the results of running privacy-preserving quantum optimization on a noisy simulator or cloud-based quantum computer.

### Success of differentially private QML

This study demonstrates that a differentially private variational quantum classifier can be trained to identify the decision boundary between two classes. Figure 10 shows that the given hyperparameters achieve good classification success. After 30 epochs, both the quantum and classical classifiers achieve accuracies greater than 95% for data organized into blobs and concentric circles. The classical network achieves 99% accuracy for the “moons” classification, but the “moons” dataset proved to be the most difficult input for the quantum classifier to classify, achieving merely 86% accuracy. It may be conjectured that the VQC had difficulties in learning the highly convex decision boundary necessary for the “moons” input set. In spite of that, the VQC generally trains just as well as a classical neural network with only 66% of the total parameters.

While DP training usually causes models to fail to capture the long tail of a data distribution, the DP-QML training is just as successful as the non-private algorithm for the “moons” and “circles” datasets, where only a modest accuracy penalty occurs for the “moons” set. Both machines can accurately classify the “circles” set without privacy, but the DP-VQC is much more successful at the task than the classical DP-NN, it even becomes more accurate, whereas the classical NN becomes less accurate under differential privacy. Our study demonstrates that quantum machine learning has the potential to reduce the loss in accuracy induced by privacy, seen in other DP applications^22,24^.

The MNIST binary classification problem creates an even more compelling case for the QML algorithm being advantageous compared to a classical ML algorithm. Table 4 demonstrates that a privacy-preserving variational quantum classifier can learn to distinguish between the handwritten digits ‘0’ and ‘1’ from the MNIST dataset to an accuracy of nearly 100%. The same table shows that a classical neural network also can accomplish the task. The greater power of the quantum model is apparent because, although the quantum network has only a quarter of the number of parameters, it achieves better accuracy than the classical neural network, as illustrated in Table 4. Furthermore, the differentially private VQC attains better accuracy than the classical neural network for $\varepsilon$’s between 0.4 and 1.4 (shown in Table 3). This range of $\varepsilon$ is sufficient, where differentially private techniques attain good privacy as defined in^22^. This work mainly focuses on the numerical demonstration of potential quantum advantages, and we leave an investigation of this from an information-theoretic perspective for future work.

**Table 4:** Results from binary MNIST classification for a $(0.406, 10^{-5})$-differentially private neural network (DP-NN) and VQC (DP-VQC).

model	DP-NN	DP-VQC
# of parameters	1029	288
Final accuracy	0.929	0.965

## Conclusion

In this work, a QML algorithm in a differentially-private framework is developed. Privacy is attained through a manipulation of the gradients of the quantum circuit. The norm of the gradient is constrained to be less than or equal to some cutoff, *S*, and then Gaussian noise is added to each gradient component, with a standard deviation of $\sigma S$. The implicit relationship between the privacy budget, *varepsilon*, and the hyperparameters, $\sigma$, *S*, and $\delta$, is provided in Eq. (14b). Overall, the QML algorithm attains the same accuracy in the MNIST classification task as the classical ML algorithm with only 28% of the number of parameters, demonstrating that the DP-QNNs are more efficient than the NN. The quantum classifier for the “blobs”, “moons,” and “circles” datasets is 66% the size of its NN counterpart and the VQC performs similarly for the “moons” and “blobs”, but better in the “circle” case. This research also shows that VQCs succeed in the privacy-preserving classification of the handwritten digits ‘0’ and ‘1’ and 2D nonlinear classifications with careful selection of hyperparameters.

This novel framework combines differentially private optimization with QML. Including DP in the algorithm ensures privacy-preserving learning. We also demonstrate a capacity for high-fidelity privacy and high accuracy in variational quantum classifiers with multiple benchmarks. Notably, for this model and dataset, we show an empirical superior performance in terms of convergence and in terms of the number of parameters of differentially-private quantum machine learning over differentially-private classical machine learning. These results indicate the potential benefits quantum computing will bring to privacy-preserving data analytics. While the questions of quantum supremacy in machine learning is still open, this study demonstrates a model and dataset, for which the QNN is more powerful than the classical model.

## Supplementary Information

Supplementary Information.

## Data Availability

The datasets used and/or analysed during the current study available from the corresponding author on reasonable request.
