# Supplementary material for: Quantum machine learning with differential privacy
Source: Sci Rep. 2023 Feb 11;13:2453. doi: 10.1038/s41598-022-24082-z (PMC9922308; doi:10.1038/s41598-022-24082-z)
Supplement: Supplementary file 1 — Supplementary Information. [file 41598_2022_24082_MOESM1_ESM.pdf]

Appendix A: Software Packages

1. Privacy Loss Calculator

The package used in this research, `PyVacy`, implements a privacy loss calculation based on the TensorFlow privacy calculator [1]. First, the Renyi differential privacy (RDP) epsilon and order are calculated. Then, the RDP loss is determined. Usually, this is more feasible because a composition property holds for (α, ε) RDP (shown in Proposition 1 of [2]). Thus, the Renyi divergences can be exactly added together given the same order. At the end of the calculation, the (α, ε) RDP is converted into the (ε, δ)-differential privacy loss. (α, ε)-RDP is defined by the Renyi divergence [2]:

$$\mathrm{D}_\alpha \equiv \frac{1}{\alpha - 1} \ln E_{x \sim Q} \left(\frac{P(x)}{Q(x)} \right) . \tag{A1}$$

The weights for the classical neural networks are initialized uniformly from the range $[-\frac{1}{\sqrt{7}}, +\frac{1}{\sqrt{7}}]$ for the first layer and $[-\frac{1}{\sqrt{2}}, +\frac{1}{\sqrt{2}}]$ for the second. For the variational quantum classifiers, the angle parameters of the unitary transforms are sampled from a normalized distribution scaled by 0.01.

2. Differentially Private Machine Learning

A micro-batch of size m and mini-batch of size n are defined, where n/m micro-batches make up a mini-batch. The loss and its gradient are calculated for each micro-batch, and a total norm of gradients is calculated. The micro-batch gradients are scaled and summed together with additional noise. This accumulated gradient now is the effective gradient for the mini-batch. This creates DP by limiting the effect each training point has on the batch gradient.

The Python package `PyVacy` is used to add DP to the RMSprop optimizer employed in this study. The DP optimizer crucially overrides the step() method and defines a micro-batch_step(). Normally, the loss and its gradients are calculated for each mini-batch. In `PyVacy`, each mini-batch is split into micro-batches. In each micro-batch, the loss and loss gradient are calculated. Then, the micro-batch step function is called, clipping the gradients. After all of the micro-batches, the step function is called to add Gaussian noise and update the parameters according to these altered gradients [1].

The mini-batch step takes the parameter gradients for each micro-batch and calculates the effective mini-batch gradients. First, the total norm of the parameters gradients, N, is calculated. Then, the scaled micro-batch gradients are added to a new parameter called the *accumulated gradient*. The gradients are scaled by a coefficient that scales down the gradients to a total norm equal to the norm cutoff, S, or, if necessary, $c = \min(\frac{S}{N+1e-6}, 1)$. The accumulated gradients add the micro-batch gradients together to create a new effective gradient for the mini-batch.

This effective gradient is scaled so the loss gradients, calculated from a given micro-batch, do not have too large norms. This creates DP by limiting the effect each training point has on the batch gradient [3]. In the overridden step method, the accumulated gradients have Gaussian noise added. The Gaussian noise is proportional to the norm cutoff and noise multiplier, S and z, respectively. The accumulated gradient then is scaled by the ratio of micro-batch to mini-batch sizes, and the micro-batch size usually is set to be 1. This leads to using the accumulated gradients in place of the original parameter gradients in the step update rule.

---

[1] C. Waites, "Pyvacy: Privacy algorithms for pytorch." `https://github.com/ChrisWaites/pyvacy`, 2019.

[2] I. Mironov, "Rényi differential privacy," *2017 IEEE 30th Computer Security Foundations Symposium (CSF)*, Aug 2017.

[3] M. Abadi, A. Chu, I. Goodfellow, H. B. McMahan, I. Mironov, K. Talwar, and L. Zhang, "Deep learning with differential privacy," *Proceedings of the 2016 ACM SIGSAC Conference on Computer and Communications Security*, Oct 2016.
